# Supplementary material for: Exploration on the Enhancement of Detoxification Ability of Zearalenone and Its Degradation Products of Aspergillus niger FS10 under Directional Stress of Zearalenone
Source: Toxins (Basel). 2021 Oct 12;13(10):720. doi: 10.3390/toxins13100720 (PMC8537726; doi:10.3390/toxins13100720)
Supplement: Supplementary file 1 [file toxins-13-00720-s001.zip › toxins-1345399-Supple.pdf]

Supplementary Materials: Exploration on the Enhancement of Detoxification Ability of Zearalenone and its Degradation Products of *Aspergillus niger* FS10 under Directional Stress of Zearalenone

Jian Ji, Jian Yu, Yang Yang, Xiao Yuan, Jia Yang, Yinzhi Zhang, Jiadi Sun and Xiulan Sun

Table S1. The sequencing results were compared and analyzed on the NCBI website (https://blast.ncbi.nlm.nih.gov/Blast.cgi) to identify the species of the samples submitted for inspection. Generally, the species with the homology of more than 97% and the highest homology obtained by the comparison on the NCBI is judged as the species of the submitted sample.

Detection method	Homology	Reference Species
CSQ	696/703(99%)	*Aspergillus niger*

TGCCCCCCGGAATACCAGGGGGCGCAATGTGCGTTCAAAGACTCGATGATTCACTGAATTCTGCAATTCACA
TTAGTTATCGCATTTCGCTGCGTTCTTCATCGATGCCGGAACCAAGAGATCCATTGTTGAAAGTTTTAACTGAT
TGCATTCAATCAACTCAGACTGCACGCTTTCAGACAGTGTTCGTGTTGGGGTCTCCGGCGGGCACGGGCCCGG
GGGGCAGAGGCGCCCCCCCGGCGGCCGACAAGCGGCGGGCCCGCCGAAGCAACAGGGTACAATAGACACG
GATGGGAGGTTGGGCCCAAAGGACCCGCACTCGGTAATGATCCTTCCGTTAGGGGAACCTGCGGAAGGATCA
TTACCGAGTGCGGGTCCTTTGGGCCCAACCTCCCATCCGTGTC**TATTGTACCCTGTT**GCTTCGGCGGGCCCGC
CGCTTGTCGGCCGCCGGGGGGGCGCCTCTGCCCCCCGGGCCCGTGCCCGCCGGAGACCCCAACACGAACACT
G

Table S2. Specific values of degradation rate of each generation.

Generation	Degradation Rate(%) (Mean±SD)
Raw (FS-10)	83.51±1.78
G1	80.25±1.68
G2	70.36±5.06
G3	80.56±5.56
G4	92.22±0.88
G5 (ZEN-S-FS10)	95.58±0.39

Table S3. Fitting results of first order degradation kinetics.

Concentration (ng/mL)	k_1 (h^{-1})
493.54	0.0033
481.49	0.0047
445.34	0.0096
361.01	0.0204
264.62	0.0318
107.99	0.0639

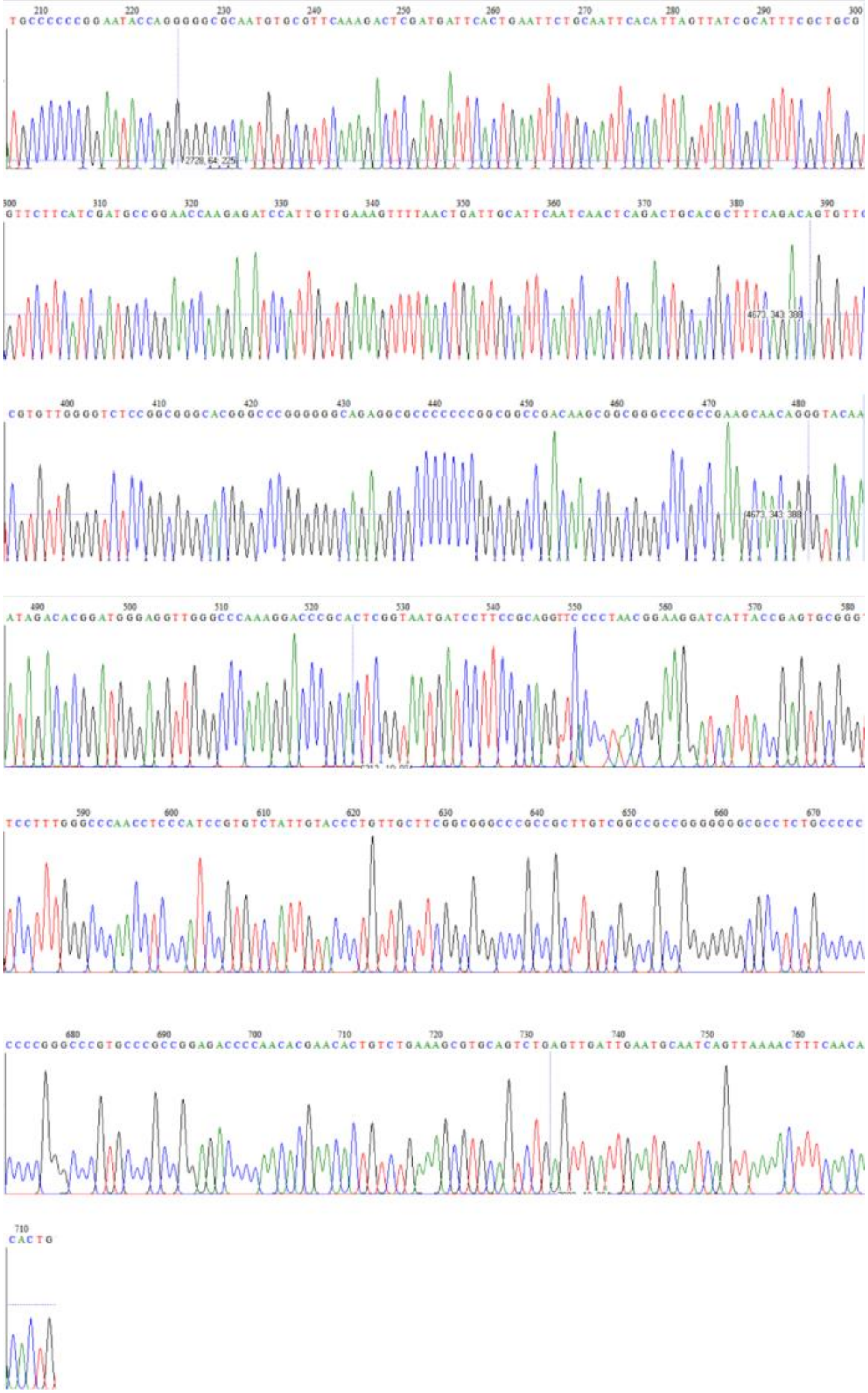

Figure S1. The content intensity of all base sequences of ZEN-S-FS10.

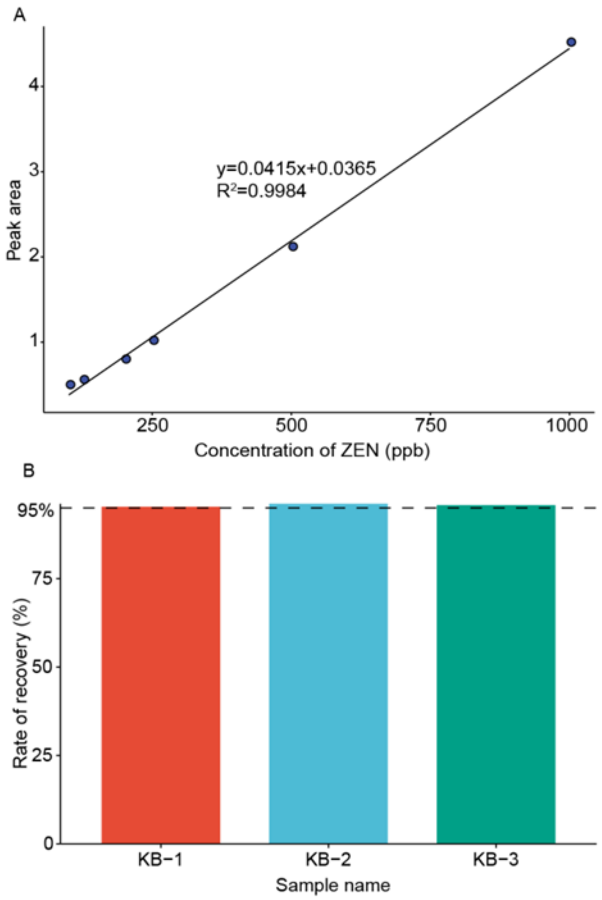

Figure S2. Recovery of ZEN in the blank control group of ZEN-S-FS10; (**A**) ZEN concentration and ration standard curve; (**B**) validation of recovery rate of blank control group.

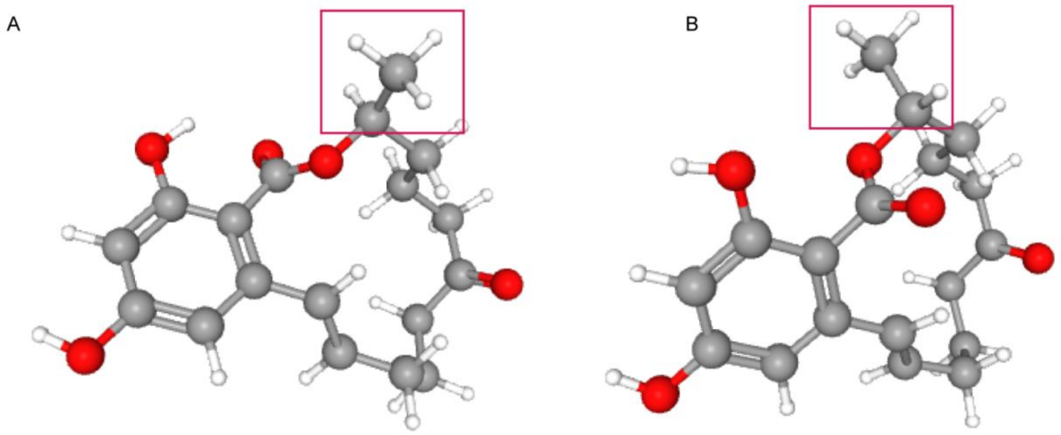

Figure S3. Comparison of the space structure of ZEN molecule and product B; (**A**) ZEN molecular space structure; (**B**) product B molecular space structure.
